# Intestinal Health of Pigs Upon Weaning: Challenges and Nutritional Intervention

**DOI:** 10.3389/fvets.2021.628258

**Published:** 2021-02-12

**Authors:** Lan Zheng, Marcos Elias Duarte, Ana Sevarolli Loftus, Sung Woo Kim

**Affiliations:** Department of Animal Science, North Carolina State University, Raleigh, NC, United States

**Keywords:** feed additives, intestinal health, newly weaned pigs, nutritional intervention, weaning stress

## Abstract

The primary goal of nursery pig management is making a smooth weaning transition to minimize weaning associated depressed growth and diseases. Weaning causes morphological and functional changes of the small intestine of pigs, where most of the nutrients are being digested and absorbed. While various stressors induce post-weaning growth depression, the abrupt change from milk to solid feed is one of the most apparent challenges to pigs. Feeding functional feed additives may be viable solutions to promote the growth of nursery pigs by enhancing nutrient digestion, intestinal morphology, immune status, and by restoring intestinal balance. The aim of this review was to provide available scientific information on the roles of functional feed additives in enhancing intestinal health and growth during nursery phase. Among many potential functional feed additives, the palatability of the ingredient and the optimum supplemental level are varied, and these should be considered when applying into nursery pig diets. Considering different stressors pigs deal with in the post-weaning period, research on nutritional intervention using a single feed additive or a combination of different additives that can enhance feed intake, increase weight gain, and reduce mortality and morbidity are needed to provide viable solutions for pig producers. Further research in relation to the feed palatability, supplemental level, as well as interactions between different ingredients are needed.

## Introduction

Weaning is considered as one of the most critical periods in pig management. It is associated with environmental, social, and dietary stress (1–3), and those various stressors result in low feed intake, body weight loss, and a high incidence of diarrhea, which consequently, can lead to mortality (4, 5). Even though trends for weaning ages at large commercial farms increase to 3–4 weeks of age, pigs are naturally weaned at the age of 12–17 weeks (6, 7). Upon weaning, at typical commercial farms, pigs deal with multiple stressors due to changes such as separation from the sow, relocation with new littermates, and sudden dietary change from sow milk to solid feeds (8). Inadequate feed intake after weaning results in insufficient dietary nutrients utilization and local inflammation (9–11). As a consequence, weaning causes profound changes in the gastrointestinal tract (GIT) of pigs. Intestine is a major site of nutrient digestion and absorption. Intestinal disorders after weaning are caused by alterations in architecture and functions with villus atrophy and crypt hyperplasia and increase in intestinal permeability (12). Moreover, intestinal microbiota disruption and changes are possibly linked to diarrhea and pathogenic infections after weaning (13–16).

Increased research needs and interests in understanding intestinal health in pigs are well-reflected in the number of peer reviewed papers searchable in PubMed (using intestinal health in pigs as keywords in the title or abstract). Since 1960 and until 2005, there have been <10 papers searched in PubMed, which has been 10 folds increased by 2018 and then 180 papers in 2020. This review focuses on feed additives as nutritional strategies to overcome weaning challenges.

## Weaning Associated Functional Changes in the Small Intestine

### Morphological Changes

**Figure 1:**
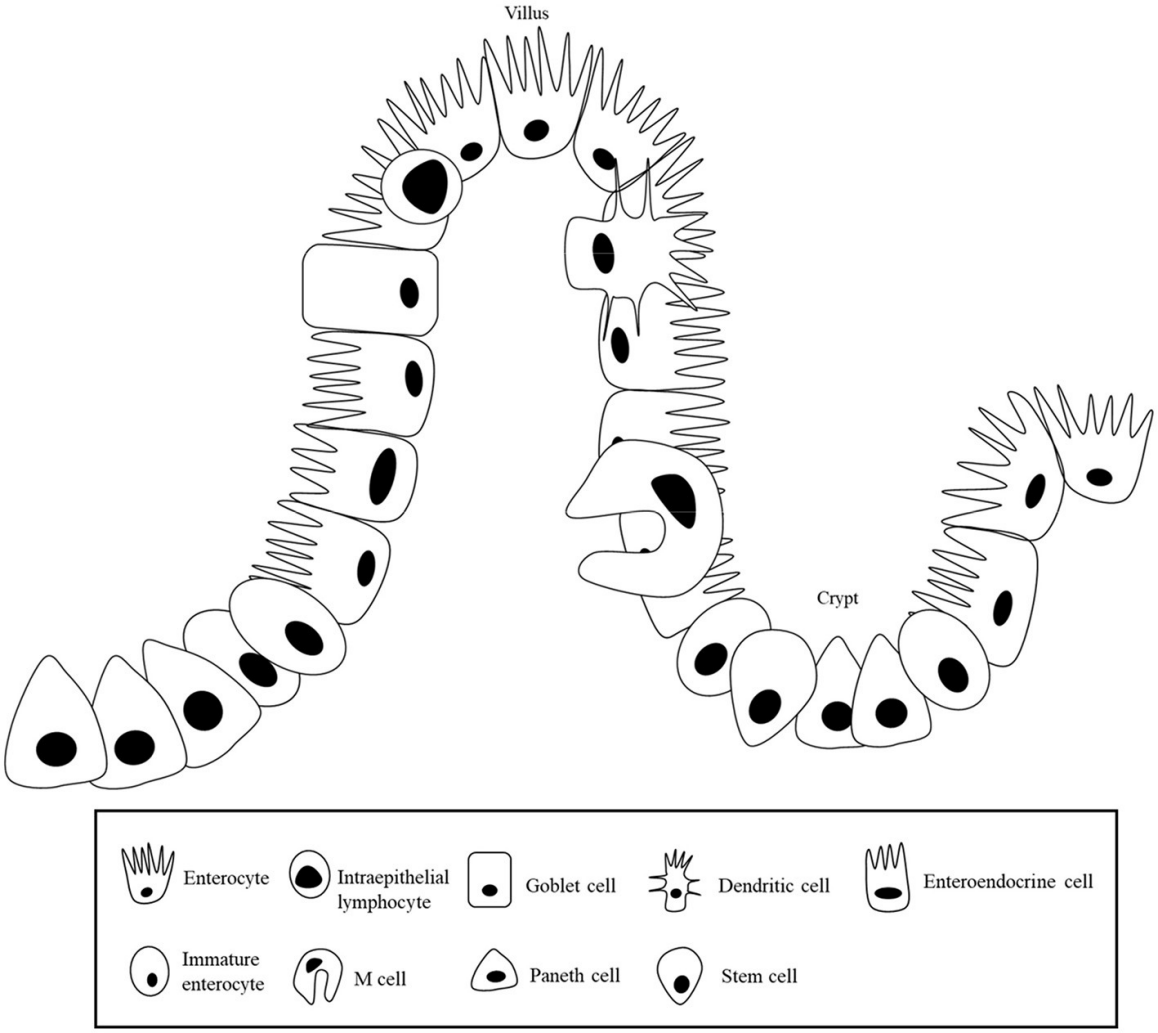
Intestinal epithelial layer.

Enterocytes are composed of villi projecting into the lumen, and a folded cell monolayer structured into crypts in pigs (17). Villi are mainly lined by enterocytes, goblet cells, and enteroendocrine cells, and the crypts are the main site containing stem cells, proliferative and undifferentiated cells, and a subset of differentiated secretory cells (Paneth, goblet and enteroendocrine cells) (18) as shown in Figure 1. When stem cells divide, they go through a cell division into a new stem cell and a committed daughter cell (19). The differentiation and maturation of each cell type happens as the cells move either migrate up the crypt–villus axis (enterocytes, mucous, and enteroendocrine cells) or downwards to the bottom of the crypt (Paneth cells) (20). In the mammalian small intestine, active enterocyte proliferation is restricted to the crypts at the base of the villi (21). Stem cells in the crypts undergo cell division and differentiation to form mature absorptive enterocytes, mucus- producing goblet cells, and enteroendocrine cells, and those cells migrate toward the villus tip, where they are discarded into the intestinal lumen (22).

After weaning, a consistent series of intestinal alterations occur. Architectural alterations associated with weaning reported in previous studies are presented in Table 1. Within 24 h of weaning, villus height was shown to reduce by 75% compared to pre-weaning status (5). The height reduction of villi is a result of increased cell loss and/or reduced crypt cell production (5). The villus atrophy and the reduction in crypt cell production during the post-weaning period result in loss of mature enterocytes, which could cause a decrease in nutrient absorption (26, 28, 29). Reduced activity of brush-border enzymes, such as lactase and peptidases and nutrient transporters, have been observed to be correlated with shortened villus height (30, 31).

**Table 1:** Morphological changes in the small intestine of pigs after weaning.

**Weaning age (day)**	**Intestinal section**	**Results**	**References**
21	Small intestine	Decreased villus height and increased crypt depth during day 11 post-weaning	(5)
21 or 35	Jejunum	Decreased villus height during day 3 post-weaning when weaned at 21 or 35 day	(23)
14	Small intestine	Decreased villus height to crypt depth ratio at day 7 post-weaning	(24)
28	75% of small intestine	Increased crypt depth at day 5 post-weaning	(25)
26	Small intestine	Decreased villus height at day 2 and 4 and decreased villus height to crypt depth ratio at day 2 and 4 post-weaning	(12)
29	Jejunum	Decreased villus height from day 2 post-weaning with minimal length was observed at day 3 post-weaning and increased crypt depth at day 5 post-weaning	(26)
21	Jejunum	Decreased villus height from day 2 post-weaning and increased crypt depth from day 5 post-weaning.	(27)

### Barrier Function

Tight junction proteins between epithelial cells form the barriers, which closes the paracellular space between epithelial cells regulating permeability through the epithelial layer (32). These proteins consist of transmembrane proteins such as occludin and claudins, as well as cytoplasmic proteins such as zonula occludens (ZO) (33). As a barrier between the luminal and basolateral compartments, tight junction proteins control the passive diffusion of ions and other small solutes, through the paracellular pathway (34). These tight junction proteins serving as a filter to allow important dietary nutrients, electrolytes, and water to translocate from the lumen of the intestine into circulation (35–37). Increases in intestinal permeability can result in inflammatory responses by allowing the entry of toxins, allergenic compounds, or bacteria (38, 39). Intestinal barrier function can be compromised by various factors, such as age, diet, pathogens, and diseases (40, 41).

Weaning induced impaired barrier function of epithelial cells promotes the entering of pathogenic bacteria and allergenic compounds from the lumen into the body (12, 42). Weaning causes compromised paracellular barrier function (2, 43). Active absorption decreases when pigs are weaned at 3 weeks of age or earlier as a process of natural intestinal maturation stimulated by weaning (Table 2); however, if pigs are weaned after 3 weeks of age, the active absorption is no more affected by weaning indicating weaning at an early age can disrupt barrier function (43).

**Table 2:** Impact of weaning age on intestinal health^a^.

**Parameter**	**Weaning age (day)**	**Experimental period (day)**	**Early weaning impact**	**References**
Morphology	21 vs. 28	56	ND	(44)
	18 vs. 20	4	↓ Villus height when challenged with ETEC	(45)
	15, 18 vs. 23	35	↑ Lamina propria cell counts	(46)
	28 vs. 49	7	↓ Villus height	(47)
Barrier function	21 vs. 28	56	↑ Expression of tight junction proteins in the jejunum	(44)
	18 vs. 20	4	↓ TER when challenged with ETEC	(45)
	15, 18 vs. 23	35	↓ TER and ↑ mucosal-to-serosal flux of mannitol and inulin	(46)
	28 vs. 49	7	↑ Mucosal-to-serosal transport of horseradish peroxidase	(47)
Mucosal immunity	21 vs. 28	56	ND	(44)
	18 vs. 20	4	↑ Mast cell activation when challenged with ETEC in pigs weaned at day 20, but not at 18	(45)
	15, 18 vs. 23	35	↑ Numbers of mast cells, corticotrophin-releasing factor, and cortisol levels	(46)
Microbiota	14, 21, 28, vs. 42	7	↓ Microbial diversity and richness	(48)

a *ND, no differences were observed; ETEC, Enterotoxigenic F18 E. coli; TER, transepithelial electrical resistance*.

### Mucosal Immunity at Weaning

Up to 70% of the immune cells are localized in the mucosa and submucosa of the intestine (49, 50). The gut-associated lymphoid tissue (GALT) consists of both isolated and aggregated lymphoid follicles forming Peyer's patches (PP) and mesenteric lymph nodes (51). The induction of intestinal immune reactions starts with antigen presentation by microfold cells (M cells) (52). Lamina propria serves as a mucosal compartment for the regulation of immune responses (predominantly IgA), with few T-cells or dendritic cells, but with myeloid cells with the characteristics of macrophages and granulocytes (53). The production of secretory antibodies, mostly IgA and IgM, is the major defending characteristics of the mucosal immune system. These antibodies are actively transported by immature epithelial cells in the crypts, and immune exclusion is carried out by the generated in cooperation with innate non-specific defense mechanisms (54). Two important periods of maximum exposure to antigens occur immediately after birth and at weaning. At weaning, the abrupt changes in the diet and environment induce alterations in the mucosal immune response (15).

The immune system in the intestine of pigs reaches an adult-like structure at 7-week-old age (55). Conventionally, weaning of pigs is done in the range of 3–4 weeks old, when cytotoxic (CD8+) T cells are primarily absent (55). Weaning also affects the systemic development of innate and adaptive immunity mainly as a consequence of the withdrawal of milk (56). Up- regulated expression of pro-inflammatory cytokines is observed in pigs at weaning (42). Recent studies have shown that pro-inflammatory cytokines, including tumor necrosis factor-α, interferon-γ, interleukin-1β, induce disturbance in intestinal barrier and increase intestinal epithelial permeability (57–59). In addition, inflammation is often associated with intestinal oxidative stress (60, 61). Disruption of cellular redox status can cause excess production of pro-inflammatory cytokines, which could further impair intestinal function (62, 63). The appropriate development of the intestinal immune system and maintaining normal redox state are essential for optimum growth and performance of the pigs. Controlling the intestinal inflammation by the over expression of intestinal pro-inflammatory cytokines may alleviate subsequent intestinal disorders induced by the weaning stress.

### Intestinal Microbiota

In pigs, the hindgut is the major site of microbial fermentation, and the microbial population in the small intestine is less diverse than the hindgut (64). The small intestine is a major place for nutrient absorption, and microbiota present in the outer mucosal layer of the small intestine are more susceptible to dietary influence (65, 66). The small intestinal mucosa is frequently exposed to various exogenous antigens and microbial components from feed ingredients. Changes in mucosa-associated microbiota may have enormous effects on host growth and development (14, 16, 67). Most of the past studies are focused on the dietary intervention on luminal and fecal microbiota, few studies evaluated on mucosa-associated microbiota. Post-weaning dietary intervention showed a long lasting effect on mucosa-associated microbiota, but not on digesta in the small intestine (16, 66). The microbial community within the outer layer of the mucosa is closely connected with host tissues, mucosa-associated bacteria are in direct competition with substrates with the host (68). Distinct microbial populations present throughout the gastrointestinal tract due to the different physicochemical conditions and substrate availability (69, 70). The fecal microbiota is distinctly different from that of the luminal of the small intestine. The similarity index of the fecal microbiota and luminal microbiota of the large intestine was 0.75, whereas it was only 0.38 when comparing the fecal and luminal microbiota of the small intestine (69). Mucosa-associated microbiota of cecum was distinctively different from that of the digesta in the cecum (64). From the outer mucosal layer into the lumen, a rapid declining oxygen gradient exists, which generating a distinct microenvironment between mucosal tissue and lumen (71). Mucosa-associated microbiota provides a line of defense against pathogens and modulates the immune status of the host (54, 72–74). The microbiota induces production of IgA by the mucosal immune system, which is secreted into the lumen to limit bacterial colonization and prevent penetration of bacteria through the epithelial layer (54, 75–77).

At weaning, the abrupt changes in the diet and environment induce alterations in the intestinal microbiota (15, 78). During the weaning transition, a major shift in the dominant genus (*Bacteroides* to *Prevotella*) was observed (79). Yang et al. (80) compared microbiota composition of healthy and diarrheic piglets and found the diarrheic piglets had an altered competitive relationship between *Prevotella* and *Escherichia* before weaning and had lower relative abundances of five genera that play key roles in nutrient metabolism (*Bacteroides, Ruminococcus, Bulleidia*, and *Treponema*) than healthy piglets after weaning. In a similar study (81), diarrheic pigs had a lower *Bacteroidales*, the fiber-degrader family, than non-diarrheic pigs during weaning, which was considered as a biomarker of diarrhea. Reductions in *Lactobacilli* is one of the most evident change after weaning (78). It was postulated the alterations in the composition and activity of the GIT microbial community is correlated with pathogenic infections after weaning (4, 82). A lower stability of the microbial community structure was observed in the ileal digesta of weaned pigs than that of unweaned pigs (78). The intestinal bacterial community composition was shown to become stable at 6 months of age (69). Table 2 summarizes the impact of weaning age on intestinal structure and function.

## Nutritional Intervention

To assist in overcoming the weaning-associated intestinal dysfunction and depressed growth, effective dietary strategies need to be explored. Feed additives including protein hydrolysates, emulsifiers, prebiotics, probiotics, feed enzymes, nucleotides, organic acids, phytogenic feed additives, immunoglobulin-containing compounds, and/or mycotoxin deactivators are commonly used in the nursery pig diets to promote growth and intestinal health (see Table 3). The following session reviews the effects of selected feed additives.

**Table 3:** Selected feed additives targeting intestinal health of newly weaned pigs with additional references.

**Feed additive**	**Initial body weight or age**	**Feeding duration (day)**	**Observations**	**References**
Fermented soybean meal	5.5 ± 0.2 kg	28	Improved growth efficiency and reduced diarrhea	(83)
	35 day	30	Increased nutrient digestibility, and positively affected fecal microflora by increasing lactic acid bacteria and decreasing *Escherichia coli* count	(84)
	35 day	35	Increased ADG and final body weight, and reduced serum urea nitrogen, increased serum immunoglobulin (Ig) G, IgM and IgA, and increased villus height of duodenum, jejunum, and ileum	(85)
	5.97 ± 0.14 kg	15	Modulated the expression of genes related to inflammatory response and anti-oxidant activity leading to a reduction on serum cortisol after lipopolysaccharide challenge	(86)
Fermented soybean protein	5.8 ± 0.9 kg	28	Improved ADG, ADFI, FCR, and increased digestibility of dry matter, gross energy, crude protein, fat, Ca, P, and increased villus height of duodenum, jejunum, and ileum	(87)
Emulsifiers	6.0 ± 0.2 kg	14	Positively affected fat digestibility	(88)
	7.9 ± 1.0 kg	35	Increased ADG, digestibility of dry matter, gross energy, and crude fat, and decreased serum triglyceride concentration	(89)
	7.2 ± 0.1 kg	19	Increased villus height of duodenum and jejunum, enhanced barrier function and positively affected fat digestibility	(90)
Probiotics	7.7 ± 1.1 kg	21	Increased feed intake, ADG, and increased digestibility of nitrogen and phosphorus	(91)
	7.6 ± 0.6 kg	42	Improved ADG and FCR during 14-day post-weaning, increased protein digestibility, increased villus height of jejunum and ileum, and increased expression of tight junction proteins when added into a low crude protein diet.	(92)
	21 day	16	Modulated intestinal microbiota by increasing *Firmicutes* phylum in the ileum and increased *Actinobacteria* phylum which includes *Bifidobacteria* in the colon	(93)
	8.4 ± 0.2 kg	28	Microbial shifts in the porcine gut in response to diets containing *E. faecalis* were similar to the response to which containing antibiotics	(67)
Prebiotics	6.3 ± 0.3 kg	28	Increased growth efficiency, increased digestibility of dry matter and affected *Bifidobacteria* concentrations	(94)
	6.13 ± 0.13 kg	14	Selectively stimulated the number of *Lactobacilli* whereas suppressed *E. coli* and *Sreptococcus. suis* and improved intestinal barrier function	(95)
	5.65 ± 0.27 kg	21	Upregulated the expression of TLR4 and calprotectin protein alleviating inflammation in the intestine and decreased diarrhea incidence challenged with enterotoxigenic *E. coli*	(96)
	4.72 ± 0.23 kg	21	Increased apparent digestibility of crude protein, calcium, and phosphorus, and decreased the incidence of diarrhea, increased the fecal shedding of *Lactobacillus* reduced *E. coli*, and improved small intestinal morphology and enhanced the growth performance	(97)
	4.9 ± 0.3 kg	14	Reduced incidence of diarrhea when challenged with *E. coli*. K88	(98)
Synbiotics	4.8 ± 0.6 kg	24	Reduced diarrhea, and increased intestinal microbial diversity when challenged with *E. coli* K88	(99)
	7.19 ± 0.45 kg	28	Improved ADG and FCR, increased digestibility of dry matter and crude protein, and increased the fecal abundance of *Lactobacillus* spp. and reduced *Enterobacteriaceae* counts	(100)
	8.09 ± 0.25 kg	28	Modulated the microbiota by increasing *Ruminococcaceae* and *Lachnospiraceae* and decreasing *Erysipelotrichaceae* and *Prevotellaceae*. Enhanced intestinal fermentation by increasing the concentration of acetate in feces	(101)
Xylanase	10.7 ± 1.2 kg	21	Increased ADG, and digestibility of dry matter and gross energy, and reduced digesta viscosity, and reduced inflammatory response	(102)
	7.2 ± 0.4 kg	24	Enhanced growth performance and gut morphology, reduced digesta viscosity, reduced intestinal oxidative stress and the enterocyte proliferation	(103)
	7.5 ± 0.1 kg	19	Increased digestibility of gross energy and total non-starch polysaccharide by increasing the digestibility of arabinoxylan. Reduced pro-inflammatory digesta viscosity, and improved intestinal barrier function	(104)
Phytase	28 day	42	Increased ADG, ADFI, and growth efficiency, and increased digestibility of minerals	(105)
	6.27 ± 0.01 kg	35	Enhanced growth performance and feed energy efficiency	(106)
Protease	6.3 ± 0.5 kg	14	Improved ADG, ADFI, FCR, reduced diarrhea, increased digestibility of crude protein, enhanced intestinal morphology, and increased nutrient transport efficiency	(107)
	8.3 ± 0.63 kg	21	Improved growth performance and reduced fecal score. Improved digestibility of dry matter, gross energy, crude protein, and phosphorus. Reduced ammonia nitrogen in cecum and colon and total volatile fatty acid in ileum and colon. Reduced the *E. coli* and increased *Lactobacillus* count in the colon	(108)
	6.42 ± 0.12 kg	42	Enhanced growth performance and digestibility of dry matter, and nitrogen. Reduced blood creatinine and fecal NH_3_	(109)
Nucleotides	4.8 ± 0.4 kg	21	Improved ADFI, positively affected ADG, and positively enhanced villus structure	(110)
	7.3 ± 0.1 kg	28	Improved ADG and ADFI	(111)
	7.3 ± 0.3 kg	42	Increased final body weight, ADG, and growth efficiency, and increased digestibility of dry mater and energy	(112)
Organic acids	7.2 ± 0.2 kg	42	Improved ADG and FCR, increased villus height, increased acetic and propionic acid concentrations, and altered microbial community structure	(113)
	6.3 ± 0.6 kg	14	Reduced inflammatory cytokines and altered microbial community composition	(114)
	8.63 ± 1.56 kg	28	Improved ADG and FCR. Reduced diarrhea score by reducing *E. coli* count in feces. Improved digestibility of dry matter, ether extract, total carbohydrates, fiber, and phosphorus and improved intestinal morphology	(115)
Phytogenic feed additives	21 day	11	Reduced diarrhea and inflammation when challenged with *E. coli*	(116)
	7.4 ± 1.3 kg	35	Increased post-weaning feed intake	(117)
	8.4 ± 1.6 kg	35	Increased weight gain, improved fecal consistency, and increased digestibility of dry matter and crude protein	(118)
	8.2 ± 2.3 kg	22	Decreased pro-inflammatory cytokines	(119)
	25 day	42	Increased growth efficiency and increased nutrient digestibility	(120)
Blood plasma	5.5 ± 0.1 kg	14	Reduced diarrhea and decreased pro-inflammatory cytokines	(121)
	6.0 ± 0.1 kg	14	Increased growth efficiency and reduced activation of the immune system	(122)
	6.8 ± 0.1 kg	12	Improved ADG, ADFI, and growth efficiency	(10)
Mycotoxin deactivators	8.2 ± 0.1 kg	34	Reduced oxidative stress and immune activation	(123)
	9.9 kg	27	Improved body weight, ADFI, and FCR	(124)
	6.0 ± 0.3 kg	35	Improved body weight, ADG, and ADFI	(125)
	9.1 ± 0.1 kg	42	Improved body weight, and ADG. Reduced TNFα, and 8-OHdG	(126)

### Protein Hydrolysates

Protein hydrolysates are produced from a variety of protein sources by chemical, microbial or enzymatic hydrolysis to eliminate or reduce anti-nutritional factors (127). Typical protein hydrolysates used in animal diets are animal protein hydrolysates (such as salmon viscera and porcine intestines) and plant protein hydrolysates (such as soybean protein hydrolysates) (128). Through the production of protein hydrolysates, anti-nutritional factors are totally or partially hydrolyzed, which make those hydrolysates a high-quality protein source for nursery pigs (129–131). Digestion of protein is mainly completed in the small intestine (132). After weaning, decreased enzymatic activity of peptidases (aminopeptidase N and dipeptidylpeptidase IV) were detected (26). Improvements in crude protein digestibility by soy protein hydrolysates supplementation have been reported in nursery pigs (133–135). Blood plasma is a commonly used animal protein hydrolysate in nursery pig diets. It has been shown to increase growth performance (136), enhance intestinal barrier function (121), and modify intestinal immune function (122) when fed to newly weaned pigs (further information see 3.9). Additionally, some peptides derived from protein hydrolysis especially milk and soy protein possess various biological functions including antimicrobial, antihypertensive, and immunomodulatory activities (86, 128, 137, 138).

#### Soy Protein Hydrolysates

Soybean meal is one of the most commonly used ingredients in animal feed; however, digestive disturbances are often observed when it is fed to young animals especially newly weaned pigs (139–141). Soybean meal contains various anti-nutritional factors including trypsin inhibitors, lectins, indigestible carbohydrate complexes, and soybean globulins (130, 139, 142, 143). Trypsin inhibitors and lectins can be inactivated by proper heat treatment and fat extraction (140, 144). However, the presence of indigestible carbohydrate complexes, antigenic soybean globulins, and residual trypsin inhibitor limits its use in young pig diets (139, 144, 145). Glycinin and β-conglycinin, antigenic proteins, are the major anti-nutritional factors that cause allergic responses in young animals (139, 146, 147). These proteins can cause hypersensitivity that induce abnormal intestinal morphological change and diarrhea when fed to young pigs (139, 148, 149). Fermented soybean meal using microorganisms such as *Aspergillus oryzae, Bacillus subtilis*, and *L. casei* and enzyme-treated soybean meal are shown to have reduced anti-nutritional factors and increased concentrations of CP and AA than conventional soybean meal (83, 150). Through the microbial fermentation or enzymatic treatment of soybean meal, the antigenic proteins are hydrolyzed into small size peptides and the glycosidic bonds in the carbohydrate fraction in soybean meal are broken down by enzymes produced by fungus and bacteria, or by a mixture of enzymes (129, 151). Fermented and enzyme-treated soybean meal have been shown to improve growth performance and feed efficiency of nursery pigs when partially replaced conventional soybean meal in the diets (83, 84). Soy oligopeptides, a soy protein hydrolysate, was shown to improve amino acid absorption compared to an intact soy protein or corresponding amino acid mixtures in a human study (152). Amino acid absorption in the portal blood from a soy protein hydrolysate was more efficient than the constituent amino acids from an amino acid mixture and those from an intact soy protein in rats (153). In addition, enhanced intestinal morphology was observed when fed soy protein hydrolysates to nursery pigs (85, 87). Despite the improved nutritional values, the bitter taste of soy hydrolysates resulting from the hydrolysis of soy proteins has been a major problem in food applications (154, 155). The hydrophobic amino acids are shown to be involved in the bitter taste of various peptides (156). Concealed hydrophobic side chains in the interior of the protein are released with the protein hydrolysis which elucidates bitterness (157, 158). Therefore, the feed palatability testing may be necessary to ascertain if soy hydrolysates can promote growth of pigs without negatively affecting feed intake of nursery pigs.

### Emulsifiers

Animal fats and vegetable oils are commonly added to meet energy concentration in the diet. To be absorbed in the gastrointestinal tract, dietary fat has to be emulsified by detergent action of the endogenous emulsifiers (such as bile salts) and hydrolyzed by lipase into fatty acids and mono- and diglycerides. Sow's milk contains ~40% fat on a dry matter basis (159, 160); whereas, typical nursery diets include fat from 3 to 6% as a maximum level (161). Digestibility of fat from sow's milk in suckling pigs is over 90%; however, digestibility of fat from solid feed in newly weaned pigs is as low as 73% (162, 163) and increases gradually return to the preweaning level ranging from 4 to 6 weeks post-weaning (23, 164). The form of the milk fat presents as micelles and consequently aid digestion (165) by pancreatic lipase, whereas fat in solid diets is not in an easily accessible form. The synthesis of hepatic bile acid is low at weaning in pigs (166). Therefore, the emulsification process is a rate-limiting step in the digestion of dietary fat during this period.

#### Lysophospholipids

Phospholipids, nature's principal surface-active agents, performs as an excellent emulsifying agent. The main constituents of the phospholipid mixture are phosphatidylcholine, phosphatidylinositol, phosphatidylethanolamine, and phosphatidic acid (167). The majority of the phospholipid in the small intestine is derived from bile with a smaller component coming from the diet. Phospholipase A2, a pancreatic enzyme secreted in bile, hydrolyzes the ester bond at the sn-2 position of the phospholipid, yielding a free fatty acid and lysophospholipids with a different head group, which are then incorporated into micelles for subsequent absorption (168–170). On a commercial scale, lysophospholipids are often produced by the modification of soybean phospholipids (chemical or enzymatic methods) using phospholipase A2 which yields a mixture of lysophospholipids with different head groups depending on the source of the phospholipids (e.g., lysophosphatidylcholine, lysophosphatidylinositol, lysophosphatidylethanolamine, and lysophosphatidic acid) (170, 171). Hydrophilic-lipophilic balance (HLB) values are assigned to emulsifiers from 0 to 20, and higher values are assigned to those are more hydrophilic. Soybean lysophospholipids have an HLB value of 19 (172), whereas the native soybean phospholipids have values of 5 (173). In addition, lysophospholipids have been reported to involve in various biological processes such as cell growth, proliferation and differentiation mediated by specific G-protein coupled receptors (174–176). Lysophospholipids supplemented in the diet showed to increase crypt cell mitosis and enhance villus morphology in broiler chickens (177). Lysophospholipids involve in epithelial cell restitution via cytoskeletal remodeling with activation of actin filament redistribution and stress fiber formation (178). It showed to reduce mucosal damage and inflammation by increasing epithelial cell restitution when induced colitis in rats (179). In broiler chickens, lysophospholipids increased crypt cell mitosis (180), and enhanced villus morphology (177).

### Prebiotics

One of the most frequently employed product is prebiotics (181). Prebiotics has been widely used for improving beneficial microbial populations in the intestines. The definition of prebiotics was first introduced by Gibson and Roberfroid (182) as “Non-digestible food ingredient that beneficially affects the host by selectively stimulating the growth and/or activity of one or a limited number of bacteria in the colon, and thus improves host health.” This concept has been refined during the past 20 years, and the definition to date was defined by Bindels et al. (183) as “a prebiotic is a non-digestible compound that, through its metabolization by microorganisms in the gut, modulates composition and/or activity of the gut microbiota, thus conferring a beneficial physiological effect on the host.” Bindels et al. (183) indicated the metabolic benefits attributed to prebiotics do not require a selective fermentation, which was mentioned in the earlier concept. The revised definition instead focused on the concept of ecological and functional characteristics of the microbiota to be relevant for host physiology, such as ecosystem diversity, and the support of broad microbial consortia. Many studies focusing on prebiotics such as inulin, fructooligosaccharides, galactooligosaccharides, and mannanoligosaccharides, proved the link between prebiotics consumption and restoring intestinal balance (184–187). Additionally, regardless of bacterial fermentation, prebiotic oligosaccharides (such as fructooligosaccharides and galactooligosaccharides) were shown to exert an anti-inflammatory effect or have an anti-adhesive activity to inhibit binding pathogens (188, 189). Studies with fructooligosaccharides showed that supplementing with fructooligosaccharides caused a shift in intestinal microbial composition via modulating short-chain fatty acids production, which provides substrates and promotes normal proliferation and differentiation of intestinal cells (190, 191).

#### Fermented Rice Bran Extracts

Rice bran, a co-product obtained during rice milling process, is rich in cell wall materials such as hemicellulose and cellulose containing neutral detergent fiber in the range of 19–34% (192, 193). The high fiber content is a major limitation of its use in young animal diets especially in newly weaned pigs. Defatting, fermentation, and enzymatic treatment (193–195) have been applied to improve the nutritional value of rice bran. Prebiotic properties of rice bran were reported in studies with mice (196) and pigs (94). Glucooligosaccharides, one of the emerging prebiotics was shown to be assimilated by *Bifidobacterium* species, but not by pathogenic species including *Clostridium* and *Salmonella* (197). Rice bran oligosaccharides, mainly composed of glucooligosaccharides, was reported to possess prebiotic potential (193, 198). The rice bran glucooligosaccharides was shown to be able to promote the growth of *Lactobacillus* species, which was not hydrolyzed by human intestinal conditions.

### Probiotics

Probiotics is defined as “living microorganisms that, on ingestion in sufficient numbers, exert health benefits beyond basic nutrition” (199). Prebiotics and probiotics exert their beneficial effects in a similar manner, through the modulations in the intestinal microbiota. Probiotics affect the microbiota via beneficial microorganisms, whereas prebiotics alter the microbiota by the supply of a substrate. Cultures commonly used in feed are lactic acid bacteria, Bacillus and yeasts (200). The beneficial microbes play an important role in maintaining the host health. They reduce the colonization and invasion of pathogens, maintain epithelial integrity, and enhance immune function (201, 202). Probiotics used in pig diets showed beneficial effects including reduced diarrhea incidence and improved in growth performance (13, 203). The combinational use of prebiotics and probiotics as synbiotics beneficially affects the microenvironment of the intestines to improve the survival and colonization of live beneficial microorganisms in the GIT (204–206).

### Postbiotics

Postbiotics is relatively new term in animal science and collectively refers to bioactive compounds produced by probiotic microorganisms during a fermentation process (207, 208). Postbiotics, in fact, has been used in animal production in different terms including bacterial extracts and yeast culture. Postbiotics often includes microbial cell contents and cell wall. Fermentation products of *Saccharomyces cerevisiae*, also called yeast culture, have long been used in animal feeds to enhance appetite of lactating animals (104, 209–211), but more recently to enhance intestinal health of nursery pigs by bioactive compounds in fermentation products (212, 213). Yeast culture includes residual yeast cell wall fragments, and various products from yeast fermentation such as organic acids, nucleotides, vitamins, and amino acids (104). Yeast cell wall fragments have also used as postbiotics to modulate intestinal immune status and health (2, 126, 214). Selected bioactive compounds in postbiotics are proposed to alter microbiota composition (215). Selected postbiotics could also be investigated for their synergistic benefits with the use of probiotics.

### Feed Enzymes

The major goal of the use of feed enzymes is to eliminate anti-nutritional factors to better utilize nutrients in the feed (200, 216). Carbohydrase has been widely used for their roles in breaking down non-starch polysaccharides (NSP) present in most vegetable ingredients (217, 218). The use of NSP enzymes showed to improve the growth performance of nursery pigs by enhancing intestinal health, nutrient digestibility (192, 194, 195). Chen et al. (102) evaluated supplemental effects of xylanase fed to nursery pigs with or without 30% corn distillers' dried grains with solubles (DDGS) as a source of NSP. The supplementation of 30% DDGS increased digesta viscosity, reduced the digestibility of dry matter and gross energy, and increased intestinal inflammation, whereas the supplementation of xylanase alleviated the negative effects on growth performance by feeding high-level DDGS by reducing digesta viscosity, improving nutrient digestibility, and reducing inflammatory response. In addition, xylo-oligosaccharides generated in the small intestine from xylans by xylanase hydrolysis could be potential prebiotics for lactogenic bacteria which warrants further research.

Protease breaks down peptide bonds in protein and polypeptides. Specific protease can target allergenic proteins in legume seed meals, such as glycinin and β-conglycinin causing gut inflammation, diarrhea and growth reduction (108). Duarte et al. (103) and Chen et al. (219) showed supplemental protease reduced gut inflammation and improvement protein digestibility and feed efficiency in nursery pigs. Phytase catalyzes the phytate hydrolysis and releases phosphorous and phytate-bound nutrients (220). The use of phytase increased phosphorus digestibility, bone characteristics, and growth performance (105, 221). More recently elevated dose of phytase so called superdosing of phytase (often more than 10-folds of typical dose levels) has received attention and applied in pig production. It is hypothesized that typical supplementation level of phytase would not completely hydrolyze phytate in the stomach and superdosing of phytase would provide opportunities of complete hydrolysis of phytate in the stomach. Complete hydrolysis of phytate not only provides available phosphates along with release of other essential minerals but also free inositol for their potential function in insulin sensitivity and carbohydrate metabolism.

### Nucleotides

Nucleotides are bioactive molecules that play important roles in metabolic, structural and regulatory functions (222). The milk of sow contain large concentration of nucleotides during 28-day lactation (223) that supplies the needs of the piglets. At weaning, the requirement of nucleotides increases for immune response and the intestinal recovery, whereas the endogenous synthesis is insufficient to meet the requirements (224, 225) and the weaning diet has low concentration compared with milk (226). Therefore, exogenous sources of nucleotides can be used to supply this demand and alleviate the effects of the weaning stress (110, 111, 223, 226). Sauer et al. (226) reported that dietary nucleotides positively affect the intestinal morphology, the immune response, the hepatic function and the microbiota. The consumption of nucleotides can improve the feed efficiency of nursery pigs by reducing the immune response and the oxidative stress status, whereas increasing the villus height and the energy digestibility (110, 111). The effect of dietary nucleotide on modulating the immune system and the microbiota suggested that it can be used to prevent post-weaning diarrhea in pigs as confirmed by Wiseman (225). According to Li et al. (112) dietary nucleotides can reduce diarrhea caused by enterotoxigenic *E. coli* by modulating the microbiota and enhancing the immune response of weaning pigs. Some of unsolved questions include the types and profiles of nucleotides for the effectiveness. Commercially available nucleotide supplements are typically obtained from yeast extracts providing combination of adenosine-5-monophosphate (AMP), cytidine-5-monophosphate (CMP), guanosine-5-monophosphate (GMP), and uridine-5-monophosphate (UMP). Some others source nucleotides from bacterial fermentation extensively including inosine-5-monophosphate (IMP). Ideal ratio among nucleotides and functional uniqueness of IMP warrant future investigations.

### Organic Acids and Acidifiers

Organic acids have been used in the pig diets to decrease gastric pH (227), prevent pathogenic bacterial growth (228), improve nutrient digestion (229), and improve growth performance (230). Gastric pH in weaned pigs ranges between 2.6 and 5.0, whereas the optimum gastric pH for vegetable protein digestion is in the range of 2.0–3.5. Inclusion of organic acids such as fumaric and citric acids are shown to have beneficial effects in newly weaned pigs (231, 232). Organic acids can modulate the intestinal microbiota by inhibiting the pH-sensitive microbial without affecting the lactic acid bacteria (233, 234). According to Ren et al. (235) 1% formic and propionic acid mixture can reduce the inflammatory response of weaning pigs challenged with enterotoxigenic *E. coli*. Current challenges with organic acids, however, are their effectiveness affecting luminal pH at a realistic supplementation level without affecting appetite or feed intake of nursery pigs. Recent advances to overcome these challenges include encapsulation or coating technologies.

### Phytobiotics and Phytogenic Feed Additives

The major biological functions of phytogenic feed additives (PFA) include improve feed palatability, stimulation of digestive enzyme secretions, microbiota modulation, antimicrobial, anti-inflammatory, and antioxidant activity (116, 117, 119, 236, 237). The PFA are reported to improve piglets' post-weaning feed intake and growth performance when added into sow diets. A mixture of phytogenic compounds (anethol, cinnamaldehyde, and eugenol) used as feed additive for sows during late gestation and lactation showed to increase post-weaning feed intake and growth rate of piglets (117). The three compounds were detected in amniotic fluid and the positive effects on post-weaning performance were attributed to the maternal exposure to the flavor of the phytogenic compounds. Li et al. (118) evaluated the effects of essential oil (a mixture of thymol and cinnamaldehyde) supplemented in feeds for nursery pigs with or without antibiotic growth promotors. The supplementation of thymol and cinnamaldehyde increased growth of pigs during 35-day post-weaning period, and the effect was similar to feeding antibiotics. In the same study, improved dry matter and crude protein digestibility were detected by the essential oil supplementation. Similar beneficial effects of PFA on nutrient digestibility in s nursery pigs were reported in other studies (120). The potential mechanisms of improving nutrient digestibility may be partially due to the stimulation of digestive enzymes activities and stimulation of bile secretion by phytogenic compounds (238). Beneficial effects on intestinal morphological changes may provide further information on promoting growth performance; however, the results obtained from different studies have not been consistent (239) where PFA reduced feed intake possibly due to strong aroma from oregano extracts. Commercial products often mask the aroma from PFA by encapsulation or coating which are practical for the feed application of PFA.

### Immunoglobulin-Containing Compounds

Under the commercial production systems, pigs are usually weaned at 3–4 weeks of age, whereas this is early stage of their life when the ability of pigs to produce immunoglobulins is not fully developed (55). The addition of immunoglobulins-containing compounds in the post-weaning diets may be beneficial. Immunoglobulin-rich product, blood plasma, has been shown to have beneficial effects on increasing post-weaning feed intake and growth rate, and reducing post-weaning diarrhea (121, 122, 240). Furthermore, in disease challenge studies with *E. coli*, blood plasma is reported to maintain intestinal barrier function, increase antibody production, and decrease pro-inflammatory cytokine expression (241, 242). In addition, supplementation of blood plasma is reported to alleviate negative impact on growth performance by feeding mycotoxin contaminated feed (10). However, despite its high nutritional value, the availability of amino acid (especially lysine) can be reduced with excessive heating treatment during manufacturing process of blood plasma (240). Additionally, increasing biosecurity concerns using blood plasma has limited its application in swine diets (24, 25).

### Mycotoxin Deactivators

Among the mycotoxins identified (~300–400), aflatoxins, fumonisins, ochratoxin A, trichothecenes such as deoxynivalenol (DON), and zearalenone are some of the mycotoxins that can significantly affect animals' health (27, 243). Impact of major mycotoxins on nursery pigs are summarized in Table 4. Previous studies have shown that young pigs are especially susceptible to trichothecenes (especially DON), and fumonisins due to their negative effects on intestines (252, 253). Consumption of DON-contaminated feed can decrease feed intake, impair intestinal barrier function, and increase intestinal inflammatory response in pigs (123, 254–256). Exposure to DON causes epithelial injuries and compromise barrier function by decreasing tight junction proteins expression and can modulate immune response by increasing the susceptibility to enteric infections (257–259). Commonly used methods include adsorbents (binding agents), enzymatic or microbial detoxification, purified enzymes, and/or “bio-protection” method using substances such as plant ingredients. Absorbents can absorb certain mycotoxins such as aflatoxin, but it does not work at the same extent to other mycotoxins. Murugesan et al. (27), in a study comparing the adsorption capacity of different commercially available mycotoxin binder products, showed that tested products have poor adsorption for DON. Alternative strategies such as enzymatic or microbial detoxification, where mycotoxins are catabolized or cleaved to less or non-toxic compounds are much more effective compared to using binding agents (27, 260). Holanda and Kim (123) reported that yeast-based detoxifiers with functional components can improve detoxifying properties in newly-weaned pigs fed DON contaminated feed (3.2 mg/kg), potentially by increasing adsorption capacity, improving immune function, and enhancing intestinal health. Fumonisins disrupt the synthesis of sphingolipids-containing cell membrane because they have a chemical structure that is similar to that of the sphingoid bases deoxysphinganine (261), key enzymes involved in sphingolipid biosynthesis (262). This dysregulation of sphingolipid biosynthesis causes accumulation of the sphingoid bases (sphinganine and sphingosine), and their metabolites (261, 263). Negative impact of fumonisins include porcine pulmonary edema, damages to gastrointestinal structure, and reduction in growth performance (254, 264, 265). In a study evaluated effects of different commercial products on mitigating fumonisins negative effects during nursery phase showed a bentonite and yeast-based product alleviated negative impact of fumonisin (50–60 mg/kg) on growth performance (124). Different regulations on maximum levels of mycotoxins for young pigs have been established by different countries; however, previous studies have shown that the contamination levels below the regulatory limits showed negative effects on growth performance and immune function (see Table 4). Furthermore, information on the regulatory limits on some of the major mycotoxins (i.e., zearalenone and ochratoxin A) and co-contamination of multiple mycotoxins are not available. The co-contamination with multiple mycotoxins in feed can cause more adverse effects than a single mycotoxin due to the additive or synergistic interaction (266). Additionally, limited practice on mitigating chronic exposure to low-dose mycotoxins may negatively impact production efficiency. Understanding the prevalence of mycotoxins in the feed and applying effective interventions are critical to ensure young pigs' health.

Table 4Impact of mycotoxins on nursery pigs and regulatory limit of major mycotoxins.

**Table:** 

**Initial body weight or age**	**Mycotoxin type and contamination level**	**Experimental period (day)**	**Impact**	**Reference**
11.4 ± 0.1 kg	Aflatoxins - 140 or 280 μg/kg	28	Decreased weight gain and altered humoral and cellular immune responses	(244)
14.2 ± 3.0 kg	Aflatoxins - 250 or 500 μg/kg	70	Reduced ADG and ADFI	(245)
27 day	Deoxynivalenol - 3.2 mg/kg	34	Reduced ADG during the last 13 day	(123)
10.3 ± 0.2 kg	Deoxynivalenol - 4 mg/kg	21	Reduced ADG, ADFI, and growth efficiency	(246)
8.9 kg	Fumonisins - 7.2, 14.7, 21.9, 32.7, or 35.1 mg/kg	28	Decreased ADG, ADFI, and growth efficiency increased the serum sphinganine-to-sphingosine ratio	(247)
28 day	Fumonisins - 3.7 mg/kg	28	Increased the serum sphinganine-to-sphingosine ratio and altered heart and intestine morphology	(248)
12–14 kg	Orchratoxin A - 800 μg/kg	84	Decreased BW and increased kidney weight	(249)
21 day	Zearalenone - 1 mg/kg	22	Had no effect on growth performance; however negative effect was shown on genital organs and serum hormones in gilts	(250)
10.4 ± 1.2 kg	Zearalenone - 1.1, 2.0 or 3.2 mg/kg	18	Negatively affected immune function in gilts	(251)
21 day	Aflatoxins - 180 ug/kg; Fumonisins - 9 mg/kg; Deoxynivalenol - 1 mg/kg	48	Reduced BW, ADG, ADFI, and growth efficiency	(2)
6.8 ± 0.1 kg	Aflatoxins - 2,778 μg/kg; Fumonisins - 170 mg/kg; Zearalenone - 1 mg/kg	33	Reduced ADG	(10)

**Table:** 

**Regulatory limit of major mycotoxins in finished feed of young pigs (mg/kg)^a^**					
Region	Aflatoxins	Deoxynivalenol	Fumonisins	Zearalenone	Ochratoxin A
United States	0.02	1	20	Not defined	Not defined
European Union	0.02	0.9	5	0.1	0.05

a *United States regulatory limit according to the Food and Drug Administration Regulatory Guidance for Toxins and Contaminants. European Union regulatory limit according to the European Commission Directive 2003/100/EC and the European Commission Recommendation 2006/576/EC*.

## Conclusions

At weaning, pigs deal with multiple stressors such as separation from the sow, a new environment, separation from littermates and cohabitation with new pigs, and the abrupt change of diet types from liquid sow milk to solid feeds. Weaning causes morphological and functional changes of the small intestine of pigs where most of the nutrients are being digested and absorbed. These changes can result in severe diarrhea and even cause mortality. In addition, due to the increasing feed safety concerns, volatile price of specialty feedstuffs, and regulatory changes on using certain feed additives (i.e., antibiotics and zinc oxide), some of the commonly used feedstuffs and additives in the nursery diets have been limited for their use. Alternative nutritional strategies aligning with these changes have been tried to combat the weaning challenges.

In order to minimize weaning-associated depressed growth, the need for developing effective nutritional strategies is critical. Functional feed additives that have a positive influence on enhancing intestinal health will aid in amelioration of the depressed growth and intestinal dysfunction associated with weaning stress. The functional feed additives such as protein hydrolysates, emulsifiers, prebiotics, probiotics, postbiotics, enzymes, nucleotides, organic acids, phytogenic feed additives, immunoglobulin-containing compounds, and mycotoxin deactivators were evaluated their roles in promoting intestinal health and growth of nursery pigs to allow better nutritional management during the crucial post-weaning period. The evaluations on how these feed additives affect the intestinal architectural structure, intestinal barrier function, mucosal immunity, and intestinal microbial community can provide valuable information to formulate optimized nursery diets. Combinational uses of these feed additives as synbiotics, could provide further benefits to nursery pigs.

## Author Contributions

All authors listed have made a substantial, direct and intellectual contribution to the work, and approved it for publication.

## Conflict of Interest

The authors declare that the research was conducted in the absence of any commercial or financial relationships that could be construed as a potential conflict of interest.
